# Improved delivery of cardiovascular care (IDOCC) through outreach facilitation: study protocol and implementation details of a cluster randomized controlled trial in primary care

**DOI:** 10.1186/1748-5908-6-110

**Published:** 2011-09-27

**Authors:** Clare Liddy, William Hogg, Grant Russell, George Wells, Catherine Deri Armstrong, Ayub Akbari, Simone Dahrouge, Monica Taljaard, Liesha Mayo-Bruinsma, Jatinderpreet Singh, Alex Cornett

**Affiliations:** 1 C.T. Lamont Primary Health Care Research Centre, Elisabeth Bruyère Research Institute, Ottawa, Ontario, Canada; 2 Department of Family Medicine, University of Ottawa, Ottawa, Ontario, Canada; 3 Institute of Population Health, University of Ottawa, Ottawa, Ontario, Canada; 4 School of Primary Health Care, Monash University, Victoria, Australia; 5 Southern Academic Primary Care Research Unit, Victoria, Australia; 6 Cardiovascular Research Methods Centre, Ottawa Heart Institute, Ottawa, Ontario, Canada; 7 Department of Economics, University of Ottawa, Ottawa, Ontario, Canada; 8 Department of Medicine, Division of Nephrology, University of Ottawa, Ottawa, Ontario, Canada; 9 Clinical Epidemiology Program, Ottawa Hospital Research Institute, Ottawa, Ontario, Canada; 10 Department of Epidemiology and Community Medicine, University of Ottawa, Ottawa, Ontario, Canada; 11 Telfer School of Management, University of Ottawa, Ottawa, Ontario, Canada

## Abstract

**Background:**

There is a need to find innovative approaches for translating best practices for chronic disease care into daily primary care practice routines. Primary care plays a crucial role in the prevention and management of cardiovascular disease. There is, however, a substantive care gap, and many challenges exist in implementing evidence-based care. The Improved Delivery of Cardiovascular Care (IDOCC) project is a pragmatic trial designed to improve the delivery of evidence-based care for the prevention and management of cardiovascular disease in primary care practices using practice outreach facilitation.

**Methods:**

The IDOCC project is a stepped-wedge cluster randomized control trial in which Practice Outreach Facilitators work with primary care practices to improve cardiovascular disease prevention and management for patients at highest risk. Primary care practices in a large health region in Eastern Ontario, Canada, were eligible to participate. The intervention consists of regular monthly meetings with the Practice Outreach Facilitator over a one- to two-year period. Starting with audit and feedback, consensus building, and goal setting, the practices are supported in changing practice behavior by incorporating chronic care model elements. These elements include (a) evidence-based decision support for providers, (b) delivery system redesign for practices, (c) enhanced self-management support tools provided to practices to help them engage patients, and (d) increased community resource linkages for practices to enhance referral of patients. The primary outcome is a composite score measured at the level of the patient to represent each practice's adherence to evidence-based guidelines for cardiovascular care. Qualitative analysis of the Practice Outreach Facilitators' written narratives of their ongoing practice interactions will be done. These textual analyses will add further insight into understanding critical factors impacting project implementation.

**Discussion:**

This pragmatic, stepped-wedge randomized controlled trial with both quantitative and process evaluations demonstrates innovative methods of implementing large-scale quality improvement and evidence-based approaches to care delivery. This is the first Canadian study to examine the impact of a large-scale multifaceted cardiovascular quality-improvement program in primary care. It is anticipated that through the evaluation of IDOCC, we will demonstrate an effective, practical, and sustainable means of improving the cardiovascular health of patients across Canada.

**Trial Registration:**

ClinicalTrials.gov: NCT00574808

## Background

The impact of cardiovascular disease (CVD) can be reduced by addressing key risk factors including smoking, obesity, and hypertension [1-3]. Primary care is central to the prevention and management of CVD. Ninety-five percent of Canadians with chronic disease have a regular family physician [4,5]. A majority of people perceive their primary care providers as a credible resource for health information and value their advice [6,7]. Primary care visits provide a unique opportunity to monitor patients' cardiovascular health and to initiate lifestyle changes and preventive care [8-10]. Unfortunately, most primary care practices are still transitioning from approaches that are designed to treat acute illnesses and are struggling to engage in high-quality management of chronic conditions such as CVD [11-13]. There exist significant care gaps, with recent studies showing that less than half of patients with diabetes have optimal blood glucose levels [14], and only 20% of patients with dyslipidemia are being actively treated [15]. In addition, despite the fact that smokers are two to four times more likely to develop coronary heart disease [16], a 2005 report released by the Canadian Tobacco Use Monitoring Survey indicated that only 54% of smokers who visited a healthcare provider in the study year received smoking cessation advice [17].

**Figure 1:**
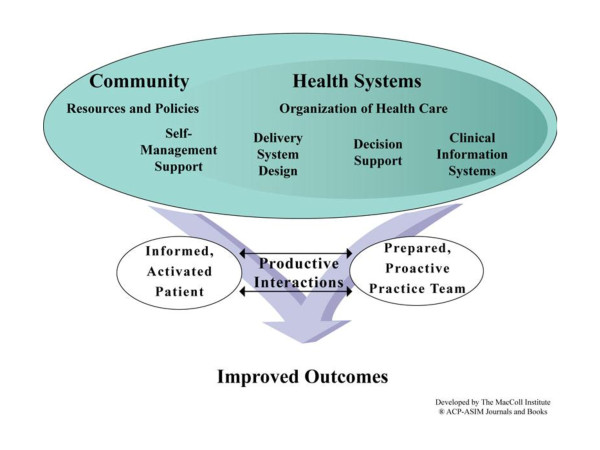
**The chronic care model, as described by Wagner et al, **[13]**identifies six essential elements for appropriate care of people with chronic diseases: **1) community linkages, 2) health care organization, 3) delivery system redesign, 4) clinical information systems, 5) decision support, and 6) self-management support for patients. Taken collectively, these six elements are intended to produce effective interactions between proactive prepared practice teams and informed activated patients who take an active part in their care. (Image from http://www.improvingchroniccare.org).

The Improved Delivery of Cardiovascular Care (IDOCC) through Outreach Facilitation project aims to improve the delivery of evidence-based care for the prevention and management of CVD in primary care practices through the use of practice outreach facilitation. The project is a multifaceted practice-tailored intervention that includes (a) audit and feedback and goal setting, (b) decision support for providers through the integration of an evidence-based cardiovascular care guideline, (c) practice delivery system redesign, (d) enhanced linkages to community resources for patients, and (e) patient self-management support tools. The intervention is delivered by a Practice Outreach Facilitator who helps incorporate these elements into daily practice routines, thus assisting physicians and staff in improving their delivery of evidence-based care for the prevention and management of cardiovascular conditions such as coronary heart disease, stroke/transient ischemic attack (TIA), peripheral vascular disease, renal failure, and diabetes. This innovative primary care quality-improvement trial is aligned with the chronic care model (Figure 1) and key findings from a recent systematic review by the Cochrane Effective Practice and Organization of Care (EPOC) Group on changing practice behavior [18]. In order to initiate positive, sustainable changes in practice behavior to improve chronic disease care, interventions should (a) be multifaceted [19], (b) be practice-tailored [20], and (c) involve system-level changes based on the elements of the chronic care model (Figure 1) [21].

Several other facilitation studies have looked at cardiovascular care [22-26], but none of these investigations have examined the impact of facilitation and its sustainability at the same depth and breadth as has the IDOCC project. Although proven to be efficacious in improving preventive care delivery in other areas of medicine, the generalizability of facilitation in a Canadian healthcare setting still remains unclear, as all investigations conducted to date have taken place in highly controlled small-scale settings, with the interventions being focused on a select group of primary care models in either urban or rural areas [27-31]. IDOCC is the first Canadian study, and one of the first studies worldwide, to examine the effectiveness of facilitation on cardiovascular care in a real life setting using a pragmatic design.

The primary research question in IDOCC is as follows: Does the large-scale implementation of a quality-improvement intervention in cardiovascular care impact (a) practice adherence to evidence-based cardiovascular care guidelines and (b) patient clinical outcomes.

This article outlines the research methods used in the IDOCC project. Unique features include the use of the stepped-wedge design, mixed methods, and the incorporation of the chronic care model approach. We hope to demonstrate that the use of practice outreach facilitation, grounded in the chronic care approach, can be successfully translated into different Canadian primary care organizations as an effective, practical, and sustainable means of improving cardiovascular health.

## Methods/design

### Overview

IDOCC is a stepped-wedge cluster randomized control trial where Practice Outreach Facilitators work with primary care practices to optimize CVD prevention and management in those patients at high risk. The multifaceted intervention, based on the chronic care model, is being offered over a 24-month period. The primary outcome is a composite score measured at the level of the patient to represent each practice's adherence to evidence-based guidelines for CVD care.

### Design

**Figure 2:**
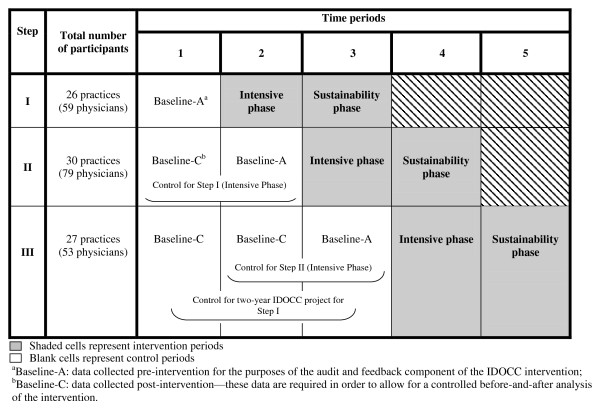
**IDOCC Step-wedge study design**.

This cluster randomized control trial employs a stepped-wedge design. A stepped-wedge design is a type of crossover study in which clusters cross over from the control arm to the intervention arm at different time points [32]. The IDOCC program is being offered to practices randomly assigned by region to one of three distinct steps (26-30 practices per step), with each consecutive step (or cohort of practices) beginning the program approximately one year apart. The intervention is delivered to practices over a two-year period (Figure 2):

1. Year 1 - Intensive Phase: Practices receive Outreach Facilitator visits every three to four weeks.

2. Year 2 - Sustainability Phase: Project intensity is lessened, with Facilitator visits every 6 to 12 weeks.

At the end of Year 2, a follow-up patient medical chart audit is used to examine changes in adherence to guidelines and patient clinical outcomes between baseline and Year 1 and baseline and Year 2 of the intervention (see section on Project Outcomes). The time offset in the rollout of the intervention allows us to control for secular changes over time as subsequent steps act as a control for previous steps. For example, when examining the impact of the intensive phase of the intervention, Step II practices will serve as controls for Step I practices, and Step III practices will serve as controls for Step II practices (Figure 2). Specific comparisons of interest in this project along with their temporal controls are presented in Table 1. Table 2 presents the progress of the IDOCC project.

**Table 1:** IDOCC study comparisons

Phase	Step	Change in outcome	Temporal/societal control
Intensive	I	X_SI, T2 _- X_SI, T1_	X_SII, T2 _- X_SII, T1_
	
	II	X_SII, T3 _- X_SII, T2_	X_SIII, T3 _- X_SIII, T2_

Sustainability	I	X_SI, T3 _- X_SI, T2_	X_SIII, T3 _- X_SIII, T2_
	
	II	X_SII, T4 _- X_SII, T3_	----

Overall study	I	X_SI, T3 _- X_SI, T1_	X_SIII, T3 _- X_SIII, T1_

SI = Step I, SII = Step II, SIII = Step III, T1 = Time Period 1, T2 = Time Period 2, T3 = Time Period 3, X = a quality-of-care outcome or a patient clinical outcome.

Note: There is no control for the Step II sustainability phase

**Table 2:** Study design and progress of IDOCC program participation of practices and physicians as of April 2011

	IDOCC program		
		
Study Design	Intensive phase	Sustainability phase	Final data collection
**Step I - First cohort of practices** **(26 practices, 59 physicians)**			

Start:	April 2008	April 2009	July 2010

Completed as of April 2011:	26 practices, 59 physicians	26 practices, 59 physicians	26 practices, 59 physicians

Projected completion:	January 2010 (Complete)	January 2011 (Complete)	February 2011 (Complete)

**Step II - Second cohort of practices** **(30 practices, 79 physicians)**			

Start:	April 2009	April 2010	October 2011

Completed as of April 2011:	30 practices, 79 physicians	1 practice, 2 physicians	--

Projected completion:	March 2011 (Complete)	March 2012	September 2012

**Step III - Third cohort of practices** **(27 practices, 53 physicians)**			

Start:	January 2010	January 2011	July 2012

Completed as of April 2011:	13 practices, 25 physicians	--	--

Projected completion:	June 2011	June 2012	June 2013

### Study setting

The project is set in primary care practices in the Champlain Local Health Integration Network (LHIN) located in Eastern Ontario, Canada. The Champlain LHIN is a culturally diverse region with a population of 1.2 million people who have chronic disease burdens and patient health outcomes that are comparable to Ontario and the rest of Canada [33]. Excluding walk-in clinics, all models of primary care practices in the selected geographic areas of the Champlain LHIN are eligible to participate in this project, including solo practices, group practices, community health centers, and academic health center teaching practices.

**Figure 3:**
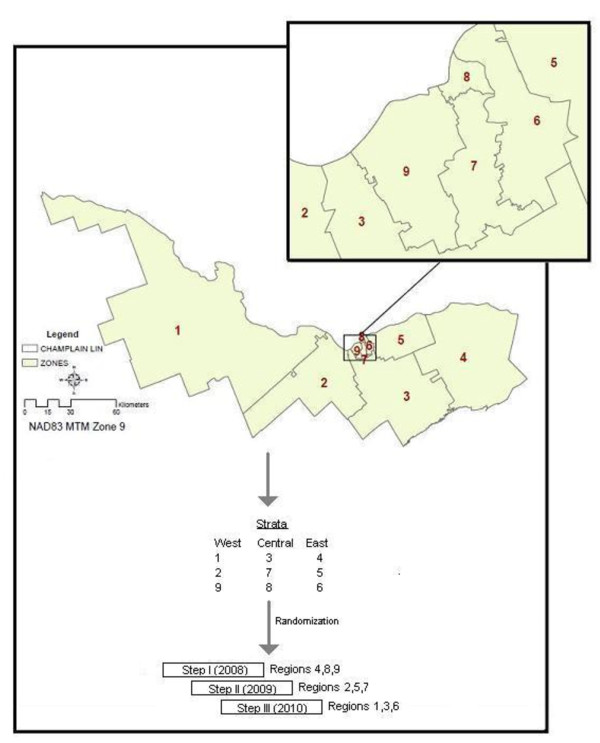
**Geographic breakdown of the Champlain LHIN**.

The Champlain LHIN was first divided into nine geographic regions using Geographic Information System mapping technology [34] and then the regions were grouped together into strata by their location (*i.e*., west, central, and east) (Figure 3). Next, a computer-generated randomization assigned each region within each stratum into one of the three steps. Randomization at the regional level was done (a) to ensure that each step was comprised of one region from each stratum (east, central, and west), (b) to ensure that each region per stratum had the same probability of being in any given step and thus the same probability of beginning the intervention at any given time point, and (c) because practice-level randomization was logistically impractical for traveling between practices randomly scattered throughout this large health region (16,000 sq. km). Using provincial administrative data, practice-level (*i.e*., practice model type, rurality, average physician age, gender mix) and patient-level (*i.e*., age, sex, socioeconomic status, number of comorbidities) characteristics of practices that consented to take part in IDOCC will be compared across the nine regions and across the three steps to assess any nonandomness of the randomization process, as well as across all practices and patients in Ontario to assess provincial representativeness.

### Practice recruitment

Prior to the initiation of the recruitment process for each step, we developed an up-to-date list of the contact information of all physicians practicing in the geographic regions of interest using a variety of physician listings, such as The College of Physicians and Surgeons of Ontario website, the yellow pages, the provincial directory of group practices and through direct contact with the practices. Practice recruitment was carried out using a modified Dillman approach involving reminders and repeat mailings [35]. While no compensation was offered to practices for participation, a pre/post-intervention chart audit measuring the quality of their practice's overall cardiovascular care and the opportunity to obtain continuing professional development credits upon completion of the program (15 Mainpro-C credits, http://www.cfpc.ca/MainproCCredits/ acted as incentives. Practices were enlisted into the IDOCC program if at least one physician from that practice agreed to participate in the project. Practices agreeing to take part in the project were asked to fill out a practice-characteristic survey highlighting details about their practice, including number of patients for the physician, primary care model type, years in practice, etc. The consent signed by each physician allowed the project team to collect information from their patient medical charts and to link this information to the province's health administrative databases. Practices entered the project immediately after consenting to the project. We also completed nonparticipant surveys for the practices that decided not to take part in the project. The purpose of the survey is to identify reasons for not wanting to participate and collect data on practice characteristics so that we could assess participant selection bias.

### Target patient population for quality improvement

The aim of the IDOCC project is to improve the quality of care delivered to those patients with and at high risk of CVD. This includes patients being over 40 years of age with at least one of the following four criteria: (1) CVD including coronary artery disease, cerebrovascular disease (documented stroke and/or TIA), or peripheral vascular disease; (2) diabetes mellitus; (3) chronic kidney disease; and/or (4) be at high risk for CVD based on the presence of at least three of the following cardiovascular risk factors: age (males ≥ 45, females ≥ 55), smoker, hypertension, and dyslipidemia [36].

### Components of the IDOCC project

Key components of the IDOCC project include using the Outreach Facilitator to deliver elements of the chronic care model to each practice (the healthcare organization of interest). The practice outreach facilitation approach includes audit and feedback, consensus building, regular meetings (monthly to quarterly) with the practices, and interactive collaborative meetings:

a) Practice outreach facilitation: Four health professionals with master's degrees and clinical and managerial experience are employed as Practice Outreach Facilitators and work with 10-15 practices each. All had previous primary care experience and underwent a seven-week intensive training course focused on quality improvement and change-management techniques, the chronic care model, and system tools for cardiovascular care, including the evidence-based guideline used by the IDOCC project http://www.idocc.ca. This training provided them with the knowledge base and hands-on training required to effectively support healthcare providers in implementing changes to their practices.

i) Audit and feedback: The chart audits are completed by trained chart abstractors who randomly select 66 target-population patient charts per practice to assess the pre-intervention performance of each practice [37]. Information is collected regarding disease/risk factor screening, prescribing, community and self-management support referrals (*e.g*., referral to smoking cessation programs), and clinical outcomes for patient groups (*e.g*., blood pressure measures, hemoglobin A1c results). The Outreach Facilitator presents these chart audit results to all primary care providers and relevant office staff in the practice during a 30-60 minute meeting using a Microsoft PowerPoint (Microsoft Corporation, Redmond, WA, USA) presentation. The practice's performance is compared to the average performance across all IDOCC project participants and published regional numbers in order to highlight both areas of high-quality care and potential areas for improvement.

ii) Consensus building and regular meetings: The Outreach Facilitator then works with the primary care providers and relevant office staff to help them identify opportunities for improvement and select appropriate strategies to address these opportunities by incorporating a chronic care model approach. Goal setting, planning, and implementation are based on the Plan Do Study Act cycle, which is a common quality-improvement tool [38]. The Outreach Facilitators regularly meet with healthcare providers over the two-year intervention period to support practices in implementing system-level changes to achieve their goals. The Outreach Facilitator supports health providers in initiating, sustaining, and measuring changes to their practice but does not take the lead in implementing these changes. Members of the practice work towards the changes they selected under the guidance and support of the Outreach Facilitator. The support includes both aiding with implementing changes and helping practices determine roles for interested team members.

Outreach Facilitators regularly meet with practice members to support change implementation and to build a trusting relationship. Relationship building has been cited as an important aspect of the facilitation process and is based on continuity of contact and the personalities of the practices and the Outreach Facilitators. Physicians participating in a past facilitation study cited the necessity of Outreach Facilitators to be available, knowledgeable, and encouraging of new ideas and involving all practitioners [39].

iii) Interactive collaborative meetings: A series of cardiovascular-care-themed half-day collaborative meetings for IDOCC participants are being held in different locations within the region. These meetings are designed to be highly interactive, as they involve group brainstorming sessions, breakout group discussions, and participants sharing experiences and approaches implemented in their practices. Topics are determined based on requests from participating physicians and, to date, have included addressing challenges in engaging patients in self-management, connecting with local pharmacists and other community resources available to patients, and diabetes care. Closely linked to the meetings are knowledge translation newsletters, which highlight particular areas for quality improvement. Newsletter topics have included smoking cessation strategies, hypertension, self-management support, and diabetes care (see http://www.idocc.ca).

b) Chronic care model: The quality improvement approach is grounded in key elements of the chronic care model (Figure 1) in order to facilitate rapid knowledge translation and practice-level changes. Outreach Facilitators focused on the following four elements: (1) evidence-based decision support for providers, (2) delivery system redesign for practices, (3) enhanced self-management support tools provided to practices to help them engage patients, and (4) increased community resource linkages for practices to enhance referral of patients. We are not specifically focusing on clinical information systems, as the majority of the practices in our health region are still paper based. The Outreach Facilitator will, however, support development and optimization of clinical information systems when identified as a need by the practice.

i) Decision support: Decision support is guided primarily by the use of an integrated CVD care guideline: The Champlain Primary Care CVD Prevention and Management Guideline http://www.idocc.ca. The goal of the Guideline is to harmonize the management and target outcomes for multiple vascular conditions (coronary artery disease, TIA/stroke, diabetes, renal failure, and peripheral vascular disease), summarize evidence-based strategies for the detection and management of these vascular conditions and their associated risk factors (high blood pressure, high cholesterol, smoking, physical inactivity, and obesity), and maximize the use of local resources and tools in the provision of care. The Guideline is a very valuable tool, as most primary care physicians struggled to follow multiple, sometimes conflicting guidelines for each individual cardiovascular condition or risk factor [10,40]. The Champlain CVD Prevention and Management Guideline is the first Canadian guideline for primary care that includes standardized care pathways for patients with multiple chronic disease and cardiovascular risk factors. It was developed based on the recommendations of seven Evidence Monitoring Committees established for each of the seven risk factors targeted by the IDOCC project: hypertension, dyslipidemia, diabetes, chronic kidney disease, smoking, obesity, and physical inactivity [41].

Upon enrollment, all practices are provided with a paper copy of the Champlain CVD Guideline in binder format and directed to the IDOCC website http://www.idocc.ca for an annually updated online version of the Guideline. At the initiation of the intervention, each Facilitator provides an overview of the Guideline to each practice team, highlighting the development, organization, and strengths of the Guideline. In subsequent visits, the Facilitator commonly refers back to the Guideline in order to stress specific evidence-based practices or to point out clinical targets and community resources.

ii) Community resources: A key feature of the Champlain CVD Prevention and Management Guideline is the community resource section in which all programs relevant to cardiovascular care for a given condition or risk factor are listed. For example, current community smoking cessation programs and exercise programs are listed with referral information specific to each region--this information is kept up to date by both the work of the Facilitators and linkages established within the Champlain CVD Prevention Network. An annual update is done on the online version of the Guideline. In cases where practices require additional information from community resources, Facilitators act as liaisons, connecting directly with education and community programs.

iii) Self-management support: Many practices are interested in enhancing self-management support within their practices. Tailored plans that are practice specific are developed to support these practices using various approaches, such as incorporating goal setting and action planning into patient visits. The IDOCC project also developed an inventory of self-management support tools such as pocket cards, flow sheets, questionnaires, and patient self-management action plan forms to help physicians improve their delivery of evidence-based care. Tools can be accessed at http://www.idocc.ca/en_toolkits.php.

iv) Delivery system redesign: In helping physicians reach their goals, the Outreach Facilitators focus on assisting practices set up new systems and processes to help improve care delivery. Specific examples of improvement strategies include the utilization of registries to track patients with certain conditions, recall systems, reminder systems, and group visits for patients with common conditions (*e.g*., several diabetes patients come in at one time to have screening tests performed and to learn about self-management). These systems are implemented into practices using a tailored approach based on practice needs and available resources. For example, some practices have electronic medical records (EMRs) that allow them to filter their patient lists to create disease-specific registries and recall systems, while other practices that use paper charts create Microsoft Excel (Microsoft Corporation, Redmond, WA, USA) documents or cue cards to track their high-risk patients.

### Data collection

The primary source of data collection consists of repeated chart audits [42] of the same randomly selected patient population from each participating practice. Chart Abstractors visit practices on two separate occasions to collect chart data--once pre-intervention and once postintervention.

### Pre-intervention

Baseline data were required to provide each practice with feedback on their pre-intervention performance levels during the audit and feedback component of the intervention (Figure 2: Baseline-A).

### Post-intervention

After Year 2 of the intervention has ended in each practice, Chart Abstractors will return to collect medical chart data from both intervention years (*i.e*., Year 1: intensive phase, Year 2: sustainability phase) and to also collect pre-intervention baseline control data (Figure 2: Baseline-C). Participation in the follow-up data collection is encouraged through the following practices: (a) practices sign a consent form stating that they will allow a post-study follow-up, and (b) each practice is told that they will receive an end-of-study progress report highlighting changes in patient health and adherence to evidence-based guidelines.

Other data sources include Facilitator narrative reports, which are essentially field notes recorded after each practice encounter, including visits, email, and telephone encounters.

### Patient selection for medical chart abstraction

Patient medical charts are randomly selected using established chart-sampling protocols utilized by the project team in the past, including the "tape measure method" for paper and mixed charting systems (*i.e*., paper and electronic records) and a random number approach for electronic records [42].

Chart Abstractors are collecting cardiovascular-related diagnostic and screening data, clinical test results, drug prescription information, and notes regarding referrals to specialists or programs. All Chart Abstractors are blinded to the exact details of the intervention and are unaware of the details of the composite score that will be analyzed at the end of this study to reduce reporting bias.

This project, including its chart audit protocol, was approved by both the Ottawa Hospital Research Ethics Board and the Bruyère Continuing Care Research Ethics Board. In accordance with the Tri-Council Policy Statement, individual patient consent was not required, since (a) the project was targeted at changing physician practice behavior within established standards of care and project staff did not work directly with the patients, (b) IDOCC will examine and report only aggregate practice population data, and (c) this project posed minimal risk to the welfare and privacy of the patients [43].

### Quality control

To ensure the consistency and quality of the abstracted data across Chart Abstractors, a four-part quality-monitoring process was established, which includes (1) standardized protocol implementation, (2) extensive data abstraction training, (3) continuous re-abstraction and validation to monitor the interrater reliability between Abstractors, and (4) constant feedback and retraining. Our overall baseline interrater reliability kappa value was 0.91, and the overall percent agreement was 94.3% [37].

### Narrative reports

Practice Outreach Facilitators are completing a narrative summary in which they document facilitation activities for each practice encounter, including face-to-face visits, telephone calls, and emails. The narratives are structured based on the chronic care model and include both long-term and short-term goals and related activities. They also include details related to challenges and barriers to change within the practice. Investigators meet on a bimonthly basis to review a sample of the narratives to monitor data quality.

### Project outcomes

Composite scores are widely employed in cardiovascular interventions targeting multiple care processes as they not only reduce sample-size requirements but also provide an overall picture of interventional benefits and group performance [44]. For this reason, a quality of care (QOC) composite score was chosen as the primary outcome for this project.

The QOC composite score reflects practice adherence to recommended process-of-care maneuvers, as outlined in the Champlain CVD Prevention and Management Guideline. The maneuvers making up the composite score are listed in Table 3 and were collected through chart audits. The QOC composite score for this project is measured at the patient level and can be summarized by the following formula:

$$\begin{array}{l} \text{QOC composite score}=\\ \frac{\sum \text{ of recommended process}-\text{of}-\text{care indicators }\text{performed}\text{ on patient}}{\sum \text{ of recommended process}-\text{of}-\text{care indicators for which the patient was }\text{eligible}} \end{array}$$

**Table 3:** List of quality-of-care indicators

Condition/risk factor	List of indicators
Coronary artery disease	➢ Blood pressure checked in past 12 months?
	➢ Fasting blood glucose taken in past 12 months?
	➢ Lipid profile taken in past 12 months?
	➢ Waist circumference measured in past 12 months?
	➢ If waist circumference is above limits (male > 88 cm, female > 102 cm), was referral to a dietician or an exercise program recommended or discussed?
	➢ Was a cardiovascular disease medication (*e.g*., beta-blocker, ACE inhibitor) recommended/discussed/prescribed in the past year?
	➢ Was aspirin recommended/discussed/prescribed in the past year?

Chronic kidney disease	➢ Albumin/creatinine ratio checked in past 12 months?
	➢ Blood pressure checked in past 12 months?
	➢ Lipid profile taken in past 12 months?
	➢ Estimated glomerular filtration rate taken in past 12 months?

Diabetes mellitus	➢ Blood pressure checked in past 12 months?
	➢ Lipid profile taken in past 12 months?
	➢ Was a glycemic control medication recommended/discussed/prescribed in past year?
	➢ Hemoglobin A1c taken twice in past year?
	➢ Waist circumference measured in past 12 months?
	➢ If waist circumference is above limits (male > 88 cm, female > 102 cm), was referral to a dietician or an exercise program recommended or discussed?

Peripheral vascular disease	➢ Blood pressure checked in past 12 months?
	➢ Fasting blood glucose taken in past 12 months?
	➢ Lipid profile taken in past 12 months?
	➢ Was a lipid-lowering medication recommended/discussed/prescribed in the past year?
	➢ Was an ACE inhibitor or angiotensin receptor blocker recommended/discussed/prescribed in the past year?
	➢ Was aspirin recommended/discussed/prescribed in the past year?
	➢ Waist circumference measured in past 12 months?
	➢ If waist circumference is above limits (male > 88 cm, female > 102 cm), was referral to a dietician or an exercise program recommended or discussed?

Stroke/transient ischemic attack	➢ Blood pressure checked in past 12 months?
	➢ Fasting blood glucose taken in past 12 months?
	➢ Lipid profile taken in past 12 months?
	➢ Was aspirin recommended/discussed/prescribed in the past year?

Dyslipidemia	➢ Lipid profile checked in past 12 months?
	➢ Was a lipid-control medication recommended/discussed/prescribed in the past year?
	➢ Waist circumference measured in past 12 months?
	➢ If waist circumference is above limits (male > 88 cm, female > 102 cm), was referral to a dietician or an exercise program recommended or discussed?

Hypertension	➢ Blood pressure checked at least three times in past year?
	➢ Was a blood pressure control medication (*e.g*., beta-blocker, ACE inhibitor) recommended/discussed prescribed in the past year?
	➢ Waist circumference measured in past 12 months?
	➢ If waist circumference is above limits (male > 88 cm, female > 102 cm), was referral to a dietician or an exercise program recommended or discussed?

Smoking	➢ Was smoking status checked in the past year?
	➢ If patient smokes, was there counselling or referral to a smoking cessation program?
	➢ Was a smoking cessation drug (e.g., Nicotine patch, Champix, etc) recommended/discussed/prescribed in the past year?

ACE: Angiotensin Converting Enzyme

### Secondary clinical outcomes

Clinical outcome data collected through the chart audit will be used to determine the change in the proportion of patients who were at target levels (as specified in the Champlain CVD Prevention and Management Guideline) for each clinical indicator upon completion of the intervention.

### Sample-size calculation

Prior to the recruitment phase of this project, a series of simulations were run on patient data from a pilot phase of the chart audit in order to examine how the primary outcome composite score varied with specific changes in physician adherence to recommended guidelines. For example, if Patient X had diabetes and a composite score of Y, how would Patient X's composite score change if he/she now had (a) a discussion about glycemic control medications with his/her physician? (b) a lipid profile test performed? (c) both maneuvers performed? The results from each simulation were discussed amongst a panel of family physicians and cardiologists in order to assess what effect size or change in composite score would represent a clinically relevant change in provider performance. From these discussions, it was agreed that an effect size (mean difference in the composite score for practices in the intervention step vs. the comparison step) of 8% represented a clinically relevant change. Using the sample-size calculation formulas for comparing two means presented by Donner and Klar [45], and assuming an intra-cluster correlation coefficient (ICC) of 0.18, a common standard deviation of 18%, and an average of 66 charts per practice, we would require 21 practices per step to detect an 8% difference using a two-sided test at the 5% level of significance with 90% power. To account for 20% potential attrition, a total of 27 practices per step were enrolled. The ICC and standard deviation used for this calculation were derived from the IDOCC pilot project on nearly 500 patients across seven practices. The anticipated attrition rate accounted for both practice-level as well as patient-level attrition and was based on experience from previous practice-based research studies [27-29].

### Data analysis

A mixed-methods approach will be used, which will consist of analysis of both quantitative data and qualitative data.

### Quantitative analysis

Descriptive statistics will be generated for all project variables (means and standard deviations for continuous variables with normal distributions or medians and interquartile ranges for skewed distributions, and frequencies and proportions for categorical variables). These statistics will be compared among the practices allocated to the different steps of the stepped-wedge design to assess imbalances in practice characteristics that would need to be controlled for in the analyses, including age, sex, practice model type, urban/rural practice setting, practice size, patient socioeconomic status (determined using a census-based method [46]), and patient comorbidities. Physicians agreeing to participate in the project will be compared to those declining to participate, and those characteristics significantly associated with participation will be adjusted for in secondary analyses to explore the potential impact of participation bias on our results. All analyses will be conducted using a commercially available software package (SAS, Version 9.2, SAS Institute, Inc., Cary, NC, USA [47]) with α = 0.05 as the level of significance.

Although the practice is the unit of intervention, all process and clinical measurements are at the patient level, and as such, the patient is the unit of analysis. All primary and secondary outcomes will be analyzed using general linear mixed-effects regression models using the stepped-wedge design analysis approach outlined by Hussey and Hughes [48]. All models include random effects for practice to account for the ICC of patients within the same practice, as well as fixed effects for region (the stratification used in the randomization scheme).

Prespecified controlled comparisons (Table 1) among practices in specific steps will be made in the regression models to examine the impact of the intervention while controlling for the effects of time. For example, in order to estimate the impact of the intensive phase of the project on Step I practices, a comparison of the patients in the intervention (Step I) and control (Step II) groups will be done by comparing the mean changes in the primary (*i.e*., QOC composite score) and secondary outcomes from baseline (Time Period 1) to the intensive phase year (Time Period 2) (Table 1).

In addition, since we are serving such a diverse group of patients with varying comorbidities, the number of indicators for which each patient is eligible varies; thus, certain patients may have a greater impact on statistical estimates of the primary outcome composite score than others. For example, if Patient A was eligible for 4 maneuvers and Patient B was eligible for 8, a change in one maneuver for Patient A results in a 0.25 change in the composite score, while the same change for Patient B only results in a change of 0.13. A "performance indicator eligibility" covariate will be included in the regression model in order to account for the differences in the number of performance indicators for which each patient was eligible.

A backwards stepwise approach will be used to establish regression models in which only variables significant at the α < 0.1 level remain.

### Qualitative analysis--Practice Outreach Facilitator narratives

Descriptive analysis will be used to report information on the types, frequency, and intensity of the Outreach Facilitator visits and interactions. Data will be prepared for analysis by entering Outreach Facilitator narratives and descriptive practice profiles into the NVivo 8 software program (QSR International, Cambridge, MA, USA). The data analysis team members will include the clinician investigators and members of the research staff trained in qualitative research.

The analysis of the data will follow standard qualitative techniques of constant comparison and immersion crystallization [49].

We will particularly explore how narratives vary between Outreach Facilitators to understand the methods they employ and investigate the degree of variation in approaches between Facilitators. We will focus on communication methods, frequency of visits, and tools used. The research staff will read and summarize the full transcripts for each practice. For the purpose of this study, we will consider each Outreach Facilitator as a separate case, with the individual practices that each Facilitator works with acting as an embedded unit of analysis. The qualitative research staff will read and reread the practice summaries and present a proposed cross-case thematic analysis to the larger analysis team including the clinical investigators for discussion. Further analyses will characterize typologies for the Outreach Facilitator roles and routines. These will be defined, discussed, and re-explored in an iterative process. Cross-case analysis will be employed to compare and contrast Outreach Facilitator methods, and these data will be triangulated with the larger datasets to determine if any of the above factors (communication methods, etc.) influence the practice's strategies for change.

## Discussion

We have described the methods for implementing a large-scale pragmatic trial in primary care. To our knowledge, the IDOCC project is the largest practice outreach facilitation trial in Canada to date. It builds on previous research in practice facilitation and attempts to translate research evidence into practice in the important area of CVD management. The use of a stepped-wedge design to evaluate this type of quality-improvement trial is novel [28,32]. The most frequent complaint made by physicians and policy makers in regards to small-scale randomized control trials and systematic reviews is that they lack widespread applicability and thus provide insufficient information to make informed decisions about translating research into practice [50,51]. For this reason, many decision makers have made a call for greater applicability in healthcare research and an increase in the implementation of "real-life" pragmatic trials [51,52].

Our real-life pragmatic study is designed so that the project can be delivered to all practice structures and primary care organizational model types. Furthermore, unlike other cardiovascular facilitation studies that have simply evaluated their interventions using self-administered questionnaires [22-25], we use a mixed-methods approach and are collecting both process implementation data and practice care delivery and patient clinical outcome data through a rigorous, stepped-wedge design. The stepped-wedge design is ideal in such a large-scale implementation project as it has the following advantages: (a) it helps overcome the practical, logistical, and financial constraints associated with large-scale project implementation; (b) it allows us to control for the effect of time as data from the intervention periods could be compared to data from the control periods of the wedge; and (c) it ensures that all practices in the project are offered the intervention [32].

Changing practice behavior requires an internal organizational change of operations best achieved through a process that integrates staff roles and responsibilities in a practice-individualized manner [18,53-56]. Practice outreach facilitation is one such method of assisting the practices to optimize care delivery. Although the effectiveness of practice outreach facilitation has been demonstrated in several randomized controlled trials [57-59] and the cost effectiveness has been proven in one trial [60], funding due to human resource costs associated with practice outreach facilitation can be challenging. The sequential rollout and also the eventual inclusion of all interested practices helped to overcome these constraints, especially in discussions with decision and policy makers. We were able to fund this project, one step at a time, through multiple partnerships with decision makers, policy makers, industry, and traditional health research funders.

Finally, IDOCC is a quality-improvement trial that is geared towards a group of related diseases, as opposed to targeting a single disease. The majority of people are affected by multiple chronic illnesses and do not identify themselves according to the disease, neither do their doctors [61]. By targeting improvements in CVD as a whole, we are addressing the complexity of caring for patients with multiple chronic illnesses in the primary care setting.

## Limitations

The greatest concern of all voluntary quality-improvement interventions, including practice facilitation, is the issue of participation bias and the idea that the physicians and practices that need help the most are the least likely to participate in quality-improvement interventions. Although it is likely that we have participation bias, we know from baseline data that a significant care gap did exist within our participating practices. We will quantify the potential participation bias within our recruitment process using data from nonparticipant questionnaires (*e.g*., physician gender, graduation year, primary care model) to compare participants versus nonparticipants to elucidate specific practice and physician characteristics that may be attributable to self-selection into this project. We also plan on linking to administrative databases to compare the baseline performance and patient characteristics (*e.g*., sociodemographic characteristics of patient population, patient comorbidities using the Charlson Comorbidity Index, number of hospitalizations, drug prescription patterns) of practices participating in the project to regional averages to see if there is a significant difference between the two. The above steps will provide insight into potential self-selection biases and the overall generalizability of our findings.

The issue of assigning weighting factors to the indicators in this project was also extensively discussed by the project team. We had considered assigning specific weights to each indicator in order to reflect their relative importance to patient health outcomes. Unfortunately, there is no empirical evidence for assigning weighting factors to the indicators in this project. Although theoretically useful, many authors have acknowledged the difficulty in effectively determining meaningful weights, and as such, few researchers use this approach [44]. Moreover, all statisticians and experts that were consulted about the primary outcome for this project advised us to move forward with the nonweighted score, as they agreed that weighted endpoints should be avoided when there is no clear evidence available to assign specific weights. In order to help avoid the problems associated with misinterpreting equally weighted endpoints, we will report the results of all components of the composite score independently to allow readers to get a clear picture of the overall impact of the intervention.

Another limitation of this study is that the timing of the intervention was randomly assigned at the regional level. A more powerful approach would have been to have random assignment at the practice level. Although ideal, practice-level randomization was logistically impractical for a project of this size, as traveling between different practices that are randomly scattered throughout this large health region (16,000 sq. km) would have greatly increased project costs and extended project timelines. By having practices grouped into regions, Outreach Facilitators and Chart Abstractors were able to visit multiple practices within a given day, something that would be difficult in a practice-randomized study.

Finally, we acknowledge the limitations of using medical chart data. Studies have demonstrated that direct observation can often identify more details about care delivery than chart audits; however, none of these studies have quantified how big this gap is [62,63]. As such, it is likely that we will underestimate the number of procedures carried out. However, numerous studies have demonstrated that chart review is the gold standard for collecting medical data as direct observation is prohibitively expensive and not feasible for a trial this large and administrative data are generally less reliable than chart data [63]. This discrepancy will affect our generalizability when we talk about the regional adherence to the Guidelines, but it should not affect the evaluation of our intervention as the same limitation applies to the data collected in all steps and all time periods.

### Implications of this research

The goal of the IDOCC intervention is to improve cardiovascular care and patient health outcomes. This project was designed to align with the chronic care model and is being delivered on a large scale to a diverse group of practice models in a real-life setting. A pragmatic, stepped- wedge design with both quantitative and process evaluations will contribute to the evidence base related to quality improvement, guideline dissemination, and cardiovascular care. The results of this pragmatic trial will inform decision makers about methods of implementing large-scale quality improvement and evidence-based approaches to care delivery. It is anticipated that through the IDOCC project, we will be able to demonstrate an effective, sustainable means of improving the cardiovascular health of Canadians.

## Competing interests

The authors declare that they have no competing interests.

## Authors' contributions

CL and WH originally conceived of and designed this study protocol. CDA also contributed to the conception of this study. GW and MT contributed to the statistical analysis plan. All other authors have contributed to the ongoing project implementation and have participated in the review and preparation of this article for publication. All authors have read and approved the final manuscript.
